# L-arginine effects on cerebrovascular reactivity, perfusion and neurovascular coupling in MELAS (mitochondrial encephalomyopathy with lactic acidosis and stroke-like episodes) syndrome

**DOI:** 10.1371/journal.pone.0238224

**Published:** 2020-09-03

**Authors:** Lance H. Rodan, Julien Poublanc, Joseph A. Fisher, Olivia Sobczyk, David J. Mikulis, Ingrid Tein

**Affiliations:** 1 Division of Neurology, Dept. of Pediatrics, The Hospital for Sick Children, University of Toronto, Toronto, Ontario, Canada; 2 Dept. of Medical Imaging, The Toronto Western Hospital, University of Toronto, Toronto, Ontario, Canada; 3 Dept. of Anesthesiology, University Health Network, University of Toronto, Toronto, Ontario, Canada; 4 Dept. of Physiology and Institute of Medical Sciences, University of Toronto, Toronto, Ontario, Canada; 5 The Toronto General Hospital Medical Research Institute, Toronto, Ontario, Canada; 6 Genetics and Genome Biology Program, The Research Institute, The Hospital for Sick Children, University of Toronto, Toronto, Ontario, Canada; 7 Dept. of Laboratory Medicine and Pathobiology, The University of Toronto, Toronto, Ontario, Canada; Universita degli Studi Magna Graecia di Catanzaro, ITALY

## Abstract

**Objective:**

We previously showed that MELAS patients have decreased cerebrovascular reactivity (CVR) (p≤ 0.002) and increased cerebral blood flow (CBF) (p<0.0026); changes correlated with disease severity and % mutant mtDNA (inversely for CVR; directly for CBF). We ran a prospective pilot in 3 MELAS sibs (m.3243A>G tRNA^Leu(UUR)^) with variable % mutant blood mtDNA to assess effects of L-Arginine (L-Arg) (single dose and 6-wk steady-state trial) on regional CBF, arterial CVR and neurovascular coupling.

**Methods:**

Patients were studied with 3T MRI using arterial spin labeling (ASL) to measure CBF and changes in % Blood Oxygen Level Dependent (BOLD) signal to changes in arterial partial pressure of CO_2_ to measure CVR. Task fMRI consisted of an alternating black and white checkerboard to evaluate visual cortex response in MELAS and controls.

**Results:**

Following L-Arg, there was restoration of serum Arg (76–230 μM) in MELAS sibs and a trend towards increasing CVR in frontal and corresponding decrease in occipital cortex; CVR was unchanged globally. There was a 29–37% reduction in baseline CBF in one patient following 6 wks of L-Arg. Pre-treatment fMRI activation in response to visual cortex stimulus was markedly decreased in the same patient compared to controls in primary visual striate cortex V1 and extrastriate regions V2 to V5 with a marked increase toward control values following a single dose and 6 wks of L-Arg.

**Conclusion:**

Proposed “healing” effect may be due to more efficient utilization of energy substrates with increased cellular energy balances and ensuing reduction in signalling pathways that augment flow in the untreated state.

**Classification of evidence:**

This prospective pilot study provides Class III evidence that oral L-Arginine (100 mg/kg single dose or 100 mg/kg three times daily po X 6 weeks) normalizes resting blood flow from elevated pre-treatment levels in patients with MELAS syndrome, selectively increases their CVR from reduced pre-treatment levels in regions most impaired at the expense of less abnormal regions, and normalizes reduced BOLD fMRI activation in response to visual cortex stimulus.

**Clinical trials.gov (NIH):**

NCT01603446.

## Introduction

Mitochondrial encephalomyopathy, lactic acidosis, and stroke-like episodes (MELAS) syndrome (m.3243A>G tRNA^Leu (UUR)^ in MT-TL1 gene) (OMIM # 590050) [1] is associated with failure to thrive, lactic acidosis, neuromyopathy, epilepsy, migraine-like headaches and recurrent stroke-like episodes (SLEs) resembling vaso-occlusive strokes [2]. These SLEs have a predilection for occipital, posterior parietal and temporal cortices [3]. However, SLEs are not restricted to vascular territories, unlike vaso-occlusive strokes [3]. They evolve subacutely over hours to days (or weeks) [4], have greater potential for reversibility [5], and have complex hemodynamic alterations [6–8]. Current literature implicates neuronal and/or glial injury due to mitochondrial failure and cerebrovascular angiopathy with dysregulated cerebral perfusion [9]. Demonstrated increases in mitochondrial size and number in cerebral vascular endothelial and smooth muscle cells support cerebral angiopathy [10–12], but cerebrovascular reactivity studies have had contradictory results [13–16].

Investigators have demonstrated a beneficial effect of L-arginine (L-Arg) therapy for the acute treatment and prevention of SLEs [17]. The rationale was to promote cerebrovascular vasodilation through endothelial nitric oxide synthase; however, blood flow dysregulation in SLEs is complex, involving both cerebral hypo- and hyperperfusion at variable timepoints [6–8], making sole vasodilatation less plausible. Serum Arg deficiency has been shown in MELAS patients at baseline, with a further decrease during SLEs [17]. Etiology of the hypoarginemia and benefit of Arg therapy, while potentially central to pathogenesis, are yet to be fully elucidated.

Blood oxygen level dependent (BOLD) functional MRI is a non-invasive means of measuring relative changes in cerebral blood flow (CBF) in response to neuronal activation, based on differences in magnetic properties of oxygenated and deoxygenated haemoglobin, between resting and activation conditions [18, 19]. Cerebrovascular reactivity (CVR) reflects the capacity of blood vessels to dilate in response to a global vasodilatory stimulus and is a marker for brain vascular reserve [20]. CVR can be measured using a BOLD sequence in combination with changes in end tidal PCO_2_. CBF can be measured using an arterial spin labeling (ASL) sequence.

We have previously demonstrated that a family of MELAS siblings had lower serum Arg (53 ± 11 μM; controls 94 ± 18; p = 0.001) with decreased CVR (p ≤ 0.002) and increased CBF (p ≤ 0.0026) compared to controls. On regional analysis, mean CVR was reduced in both frontal and occipital cortices but more so in frontal (p < 0.001) versus occipital cortex (p = 0.002). Furthermore, mean CBF was increased in both frontal and occipital cortices but more so in occipital (p = 0.001) than frontal (p = 0.0026) cortices compared to controls [21]. We further previously demonstrated that mean CBF in frontal cortex (but not in occipital infarct penumbra) correlated directly (r = +0.85 frontal) with disease severity and % mutant mtDNA whereas CVR correlated inversely (r = -0.82 frontal, r = -0.91 occipital cortex). This makes sense as the only way to increase blood flow given constant blood pressure is through vasodilation. But any vasodilation consumes vascular reserve. The degree of cerebral hyperperfusion, which translates into a reduction in flow reserve and was inversely proportional to the CVR, was thereby directly proportional to the severity of the neurological phenotype and percentage of mutant mtDNA in blood in our cohort [21]. One interpretation of MELAS cerebral blood flow physiology is that the cortical hyperperfusion may be the result of a normal flow control mechanism responding adaptively in an attempt to compensate for metabolic imbalance resulting from inefficient ATP generation from oxidative metabolism by abnormal non-vascular cerebral mitochondria or may represent a passive response to tissue acidosis or to the accumulation of other intermediary metabolites. This may support the concept that the metabolic defect and associated hyperemia in MELAS is expressed in cerebral tissue and that the hyperemia is an adaptive response to a limitation in oxidative glucose metabolism and to the reduction of high energy phosphates from the inefficient utilization of oxygen for ATP generation and/or the result of the accumulation of lactic acid or other metabolic intermediates. Thus, a compensatory increase in CBF responding to inefficient use of oxygen for ATP generation would increase blood flow and decrease vascular reserve and vascular reactivity to a vasodilatory stimulus. A second hypothesis would be that morphologically abnormal mitochondria in cerebrovascular smooth muscle and endothelial cells may lead to an angiopathy with functional impairment of blood vessel vasodilation in response to an increase in PCO_2_, thereby limiting CVR and supporting a vascular contribution to SLEs. Thirdly, hyperperfusion could be the result of both mechanisms.

The purpose of the current study was to evaluate the effects of L-Arg on CVR, cerebral hyperperfusion, and BOLD fMRI neuronal activation in our MELAS cohort to gain further insight into the mechanism (s) of its therapeutic effect.

## Materials and methods

### Classification of evidence

This prospective pilot study provides Class III evidence that oral L-Arginine (100 mg/kg single dose or 100 mg/kg tid po X 6 weeks) normalizes resting blood flow from elevated pre-treatment levels in patients with MELAS syndrome, selectively increases their CVR from reduced pre-treatment levels in regions most impaired at the expense of less abnormal regions, and normalizes reduced BOLD fMRI activation in response to visual cortex stimulus.

### Study methodology

A clinical pilot study design was used to assess the response of cerebral perfusion and cerebrovascular reactivity in MELAS patients to L-arginine. The protocol for this trial and supporting CONSORT checklist are available as supporting information; see S1 Checklist and S1 Protocol. The authors confirm that this non-randomized pilot efficacy study and all related trials for this drug/intervention were registered on the ClinicalTrials.gov (NIH) website under identifier: NCT01603446. Research ethics board (REB) approval was obtained from both study sites, the Hospital for Sick Children (HSC) and Toronto Western Hospital (TWH). A data safety monitoring committee was in place. Written informed consent was obtained from all participants and from the next of kin on behalf of all minors enrolled in the study using formal consent forms approved by the REB of the HSC. All clinical investigations were conducted according to principles expressed in the Declaration of Helsinki.

### Subjects

Three siblings (two females, one male) aged 17, 21 and 22 years with genetically confirmed MELAS syndrome (m.3243A>G tRNA^Leu (UUR)^ in *MT-TL1*) were recruited from the Neurometabolic clinic at HSC, Toronto, Canada. Four healthy age and sex-matched controls (three females, one male) living in Toronto were recruited through posted advertisements, approved by the REB, at the HSC, Toronto and the University of Toronto by referral and by self-selection. Female patients were also matched to controls for timing of menstrual cycle, and estradiol levels were measured, as estrogen appears to alter myogenic tone (vasodilation) by increasing cerebrovascular NO production and/or action [22]. Healthy controls had no ongoing medical conditions that could affect CVR (e.g. migraine, neurological disease, genetic metabolic disorder, cardiac or pulmonary disease, hypertension, prothrombotic disorder, anemia) and were taking no medications and were not smokers. Healthy controls were also screened prior to study entry for a normal baseline physical examination, blood pressure, serum hemoglobin, lactate, creatine phosphokinase, quantitative amino acids, carnitine, and urine organic acids. The patients and healthy subjects did not smoke and all subjects were asked to refrain from caffeine. The studies were performed and the data was collected at the HSC in Toronto.

### Study design

**Fig 1:**
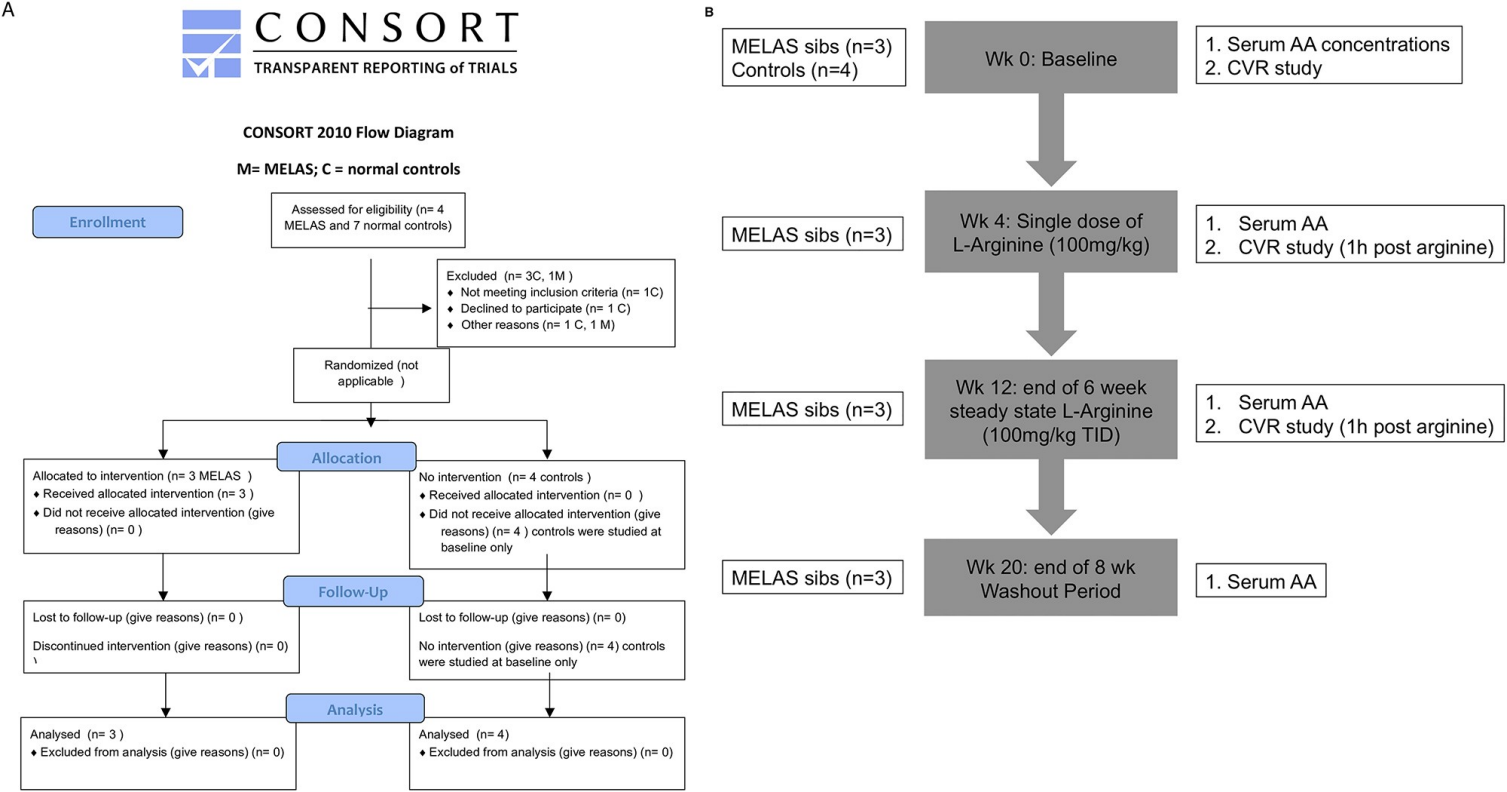
Flow diagrams for the MELAS/L-arginine study (A) Consort 2010 Flow Diagram (B) Schema for MELAS/L-arginine Study Protocol.

A Consort flow diagram is given in Fig 1A and a Study Protocol flow diagram is represented in Fig 1B. At baseline, a complete neurological examination to ensure clinical stability was done. Serum quantitative amino acids (AA), and CVR and CBF studies were performed on both MELAS patients and controls. MELAS patients also underwent baseline CBC, serum electrolytes, renal and liver functions, glucose, calcium, phosphate, PT, INR, carnitine, CK, lactate, and urine organic acids. Female subjects had serum estradiol measured. Serum AA were measured at 4 time-points over the day, both pre- and post-prandially, to better assess average AA levels. Serum arginine levels of MELAS and control subjects were compared to our well-established normal control data (n = 500) from the HSC Metabolic Diseases Laboratory using the Student`s T-test. The control females and the control male underwent only the baseline studies, which were completed in one day. Studies were conducted on separate days from the MELAS subjects. The MELAS subjects underwent the studies in tandem on the same day. Studies were conducted at 4 weekly intervals to coincide with the same timepoint in menstrual cycle (Fig 1B). On week 4, MELAS patients only were given a single dose of oral L-Arg (100 mg/kg) mixed in solution by the clinical research trial nurse in the Physiology Research Unit at the HSC, Toronto. Serum amino acids were measured following administration (4 time points: 1 hour post oral L-Arg twice and 3–5 hours post L-Arg twice), and a CVR study was performed 1 hour after administration to coincide with peak serum L-Arg concentrations. On week 6, MELAS patients were commenced on a 6 week, steady state trial of oral L-Arg at 100mg/kg three times daily as per Koga et al. [23]. At week 12 (MELAS only) serum AA were repeated (4 time points as above) along with baseline bloodwork, and the CVR study was repeated at one hour post L-Arg (100 mg/kg) administration. At week 20 (MELAS only), following an 8 week wash-out period, serum AA were repeated along with the baseline bloodwork. High grade, highly purified NOW foods commercial Natural Health Product L-Arginine Powder, NPN 80002672, Bloomingdale, Illinois, which was approved for use on the Canadian market by Health Canada, Natural Health Products Directorate, was used. All subjects received a small incentive for their participation.

### CVR studies

For CVR studies, subjects were fitted with an air-tight mask on the face attached to a sequential gas delivery breathing circuit. An automated gas blender (RepirAct^™^ Thornhill Research, Inc., ON, Canada) controlled gas delivery to the breathing circuit. In our previous study of the RespirAct^™^ sequential gas delivery circuit in five healthy male adults comparing their PETCO_2_ values with their arterial PCO_2_, repeated measures of ANOVAs revealed no significant differences between the end-tidal PCO_2_ (PETCO_2_) (between 35 to 50 mmHg), and arterial PCO_2_ (Pa,CO_2_) over the ranges of PO_2_ (between 70 to 300 mm Hg) [24]. This has been confirmed both in animals [25–27] and in humans [24, 28]. In the current study, the patterns of end-tidal (end-expiratory) partial pressure of CO_2_ (PetCO_2_) and O_2_ (PetO_2_) were programmed into the automated gas blender, which directed mixtures of O_2_, CO_2_ and N_2_ into the breathing circuit according to prospective targeting algorithms developed by Slessarev et al. [29]. Tidal gas was sampled and analyzed for PETCO_2_ and PETO_2_ and recorded at 20 Hz.

### CVR protocal

Subjects were given a series of four hypercapnic challenges where PETCO2 was raised 10 mmHg above their individual baseline for 90, 60, 60, and 60 seconds with 60 second interval returns to baseline. Total imaging time was 9.5 minutes. This was previously demonstrated to be safe, well tolerated and technically feasible in a clinical patient population ranging in age from 9 to 88 years [30]. The PETCO_2_ and PETO_2_ were precisely controlled throughout the protocol by the RespirAct^™^ and the PETO2 was maintained at 100 mmHg.

### BOLD-MRI-CVR

Magnetic resonance imaging was performed with a 3.0 Tesla Signa HDx scanner (GE Healthcare, Milwaukee, Wisconsin) using an 8-channel phased-array receiver head coil Imaging consisted of BOLD echo planar acquisitions (EPI) using a gradient echo readout (TR/TE = 2000/30 ms, 3.75 x 3.75 x 5 mm voxels, field of view 24 x 24 cm, 30 slices, slice thickness 5 mm, matrix size 64 x 64, number of frames = 254, flip angle (FA) = 85^0^). The acquired MRI and PET CO_2_ data were analyzed with AFNI software (National Institutes of Health, Bethesda, Maryland; http://afni.nimh.nih.gov/afni. To synchronize PETCO_2_ and BOLD signal data, PETCO_2_ data were time-shifted to the point of maximum correlation with the BOLD signal averaged over the whole brain. BOLD signal drift correction was made assuming a linear drift over time between the initial and final baselines [31]. Three-dimensional high-resolution T1-weighted anatomic MR images were acquired for coregistration with BOLD MR images by using an inversion-recovery fast spoiled gradient-echo sequence [30].

BOLD images were then volume registered and slice-time corrected, and co-registered to an axial 3-D T1-weighted Inversion-Recovery prepared Fast Spoiled Gradient-Echo (IR-FSPGR) volume (T1/TR/TE = 450/8/3 ms, voxel size 0.86 x 0.86 x 1.0 mm, matrix size 256 x 256, field of view 22 x 22 cm, slice thickness = 1 mm, FA = 15 ^0^) that was acquired at the same time [32] as described in Fierstra et al. [33]. All images were normalized by mapping them into the same number of voxels. This enabled the representation of the fractional frequency of CVR values by constructing frequency distribution histograms which used all CVR data with the exception of zero values [31].

The BOLD MRI signal was regressed against end-tidal CO_2_ on a voxel-by-voxel basis. The CVR was represented by the slope of the regression of the percentage change in MRI signal intensity versus mmHg change in PETCO_2_. In other words, the CVR was the % change in blood flow per unit change in flow stimulus in mmHg [31]. The slope of the relation between the BOLD signal and the PETCO_2_ was colour-coded to a spectrum of colours corresponding to the direction and the magnitude of the slope and overlaid voxel-by-voxel on precisely matched anatomical scans to generate CVR maps [31]. Each MELAS and study control subject’s CVR maps were registered in the MNI standard space and compared voxel by voxel to a CVR atlas generated from a cohort of 19 healthy subjects (ages 20–30 yrs) who had been similarly configured [34].

### Cerebral blood flow

Increases in brain blood flow occur in response to a variety of stimuli including neural activity, anemia, decreased blood pressure, and carbon dioxide. Quantitative cerebral blood flow (CBF) was measured in MELAS and control subjects at baseline using arterial spin labeling (ASL) technique (pseudocontinuous ASL product sequence, GE Healthcare, Milwaukee). The CBF was color-coded on a rainbow spectrum from 0 ml/100 g/min (blue) to 100 ml/100g/min (red). All MELAS patients and healthy controls had normal haemoglobin and hematocrit, therefore no adjustments were required for anemia. The CBF maps generated from the MRI ASL sequence were also compared to a CBF atlas of 14 normal controls (20–30 yrs) previously studied at TWH.

### Regions of interest

Given the preferential involvement of occipital cortex in MELAS SLEs, we compared CVR and perfusion that was restricted to regions of interest in the cortical grey matter of the frontal cortex (anterior circulation) and occipital cortex (posterior circulation) in our MELAS patients, study controls, and normal control population at baseline (no L-arginine). Since MELAS patient 3 had occipital gliosis from prior SLEs, and since infarcted brain has altered blood flow characteristics, we excluded regions of infarcted brain from this analysis. In order to maintain consistency, we similarly excluded a geographically matched region from the other MELAS patients, our study controls, as well as from the normal control population.

### fMRI acquisition

The fMRI stimulus consisted of an alternating black and white checkerboard pattern at a rate of 8Hz. The spatial frequency was 0.5 cycles per degree of visual angle. Luminance was set at 150 cd/m^2^ and the black and white check contrast was set at 90%. The stimulus was projected to the subject in the MRI scanner using a Resonance Technology goggle system. The stimulus followed a block design beginning with 60 rest followed by 4 alternating cycles of 15 sec of checkerboard stimulus and 30 sec rest and ending in 60 sec rest for a total imaging time of 5 minutes. Each checkerboard stimulus consisted of a nested 1 sec flash and 1 sec rest subcycle for a total of 8 subcycles since sustained flashing is not needed to maintain blood flow response to the stimulus [35]. This results in short 1 sec resting phases during the stimulus and is metabolically less stressful. Data analysis was identical to that used for BOLD CVR MRI except that a square wave representing the stimulus paradigm was used as the regressor. The BOLD signal therefore represented the neuronal response to the stimulus and the complex relationship between neuronal activity and the triggering of the hemodynamic response (termed neurovascular coupling) and the hemodynamic response itself. This study was conducted in MELAS 1 and 2 and in the four age-matched study controls only. The % BOLD signal change was measured in Brodmann visual cortical region masks including V1 (primary visual area and striate cortex) and in the extrastriate areas consisting of visual areas V2, V3, V4 and V5.

### Statistical analysis

Statistical analysis comparing MELAS patients at baseline (no L-arginine) to healthy study controls and a healthy control population and comparing CVR and cerebral blood flow in frontal versus occipital cortices in MELAS patients was conducted using an unpaired student’s t-test. Repeated measures ANOVA was used to compare mean serum ornithine, glutamate and citrulline concentrations for MELAS patients before and after single-dose and 6-week steady state L-arginine administration. Statistical significance was set at p<0.05. Pearson’s correlation coefficient (r) was determined between the % mutant mtDNA blood and the CVR and between the % mutant mtDNA blood and the cerebral blood flow respectively. The software program used for the statistical calculations was Microsoft Excel.

## Results

### Patient characteristics

The MELAS siblings shared a common genetic background. Four MELAS siblings were screened for the study and three were found to be eligible. The MELAS sisters (MELAS 1 and 2) were 22 and 21 years of age respectively and their brother (MELAS 3) was 17 years of age. Seven control subjects were screened for the study and four were found to be eligible. All study subjects completed the study. The healthy study controls were matched for age and gender (females aged 21, 21 and 22 yrs; male 17 yrs). The % mutant mtDNA in blood was 35, 41 and 59% respectively for MELAS patients 1, 2, and 3. MELAS patient 1 had a normal physical and neurological exam and good cognitive function. MELAS patient 2 had sensorineural hearing loss, peripheral neuropathy and exercise intolerance but normal peak power and a head circumference on the 3^rd^ percentile, weight less than the 3^rd^ percentile, height greater than the 10^th^ percentile. Only MELAS patient 3, who had the highest % mutant mtDNA in blood, had a history of multiple prior SLEs (left greater than right parieto-occipital) and migraine headaches. He also had a weight less than 3^rd^ percentile (height 10%) and globally reduced cognitive function with slow processing, apraxia, right hemianopsia, sensorineural hearing loss, subtle ptosis, reduced muscle bulk and tone with normal power. Brain MRI of MELAS patient 1 was normal; MELAS patient 2 had mild global cerebral cortical atrophy; whereas MELAS patient 3 had gliosis and atrophy of left greater than right occipital cortices. Magnetic resonance spectroscopy (with voxel in the occipital cortex) did not demonstrate a lactate peak for any of the MELAS patients. The healthy study controls had normal brain imaging and MRS studies with the exception of an incidental congenital variant of a retrocerebellar cyst in the 17 year old control male. The Montreal Cognitive Assessment screening tool revealed MOCA scores of 30, 26 and 10 out of 30 in MELAS patients 1, 2, and 3 respectively and 25, 30 and 28 out of 30 in the study control females (Controls C1, C2 and C3 respectively) and 28 out of 30 in the study control male (C4) (score of ≥ 26 considered as normal).

### Arginine, ornithine, glutamate, and citrulline concentrations

**Fig 2:**
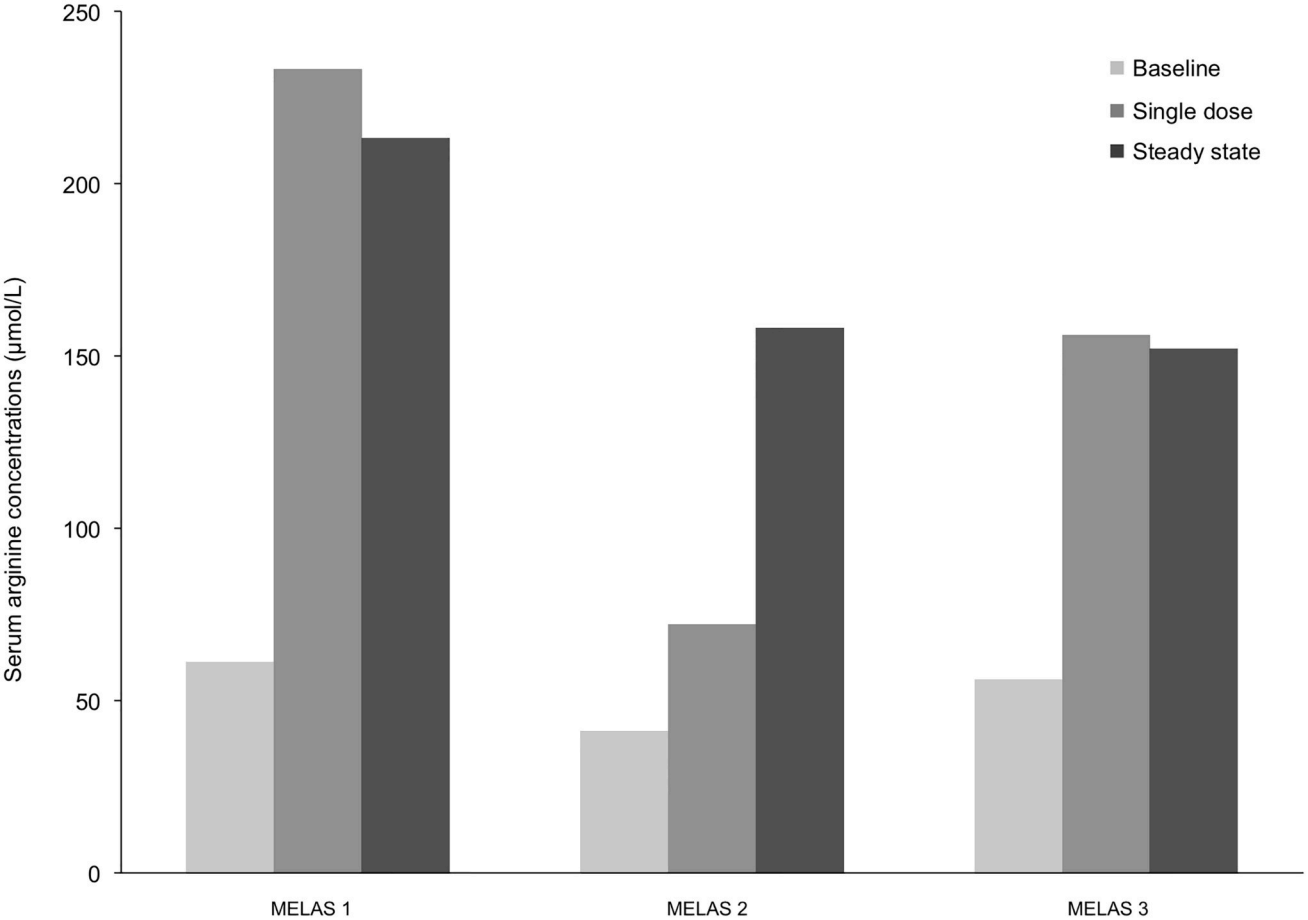
Mean serum arginine concentrations (μmol/L) at baseline and peak serum arginine concentrations following single-dose and steady-state L-arginine supplementation.

MELAS patients had significantly lower baseline serum arginine concentrations (53 ± 11 μmol/L) than study controls (94 ± 18 μmol/L; p = 0.001), although levels remained in the normal range [21]. L-Arg supplementation successfully increased serum concentrations in MELAS patients (76 to 230 μmol/L) (Fig 2). There were no significant differences between MELAS versus controls at baseline in serum ornithine (40.8 ± 12.3 μmol/L for MELAS vs 36.2 ± 7.3 μmol/L for study controls; unpaired two-tailed t-test p = 0.24) or citrulline (24.8 ± 4.8 μmol/L for MELAS vs 23.2 ± 5.4 μmol/L for controls; unpaired two tailed t-test p = 0.44) concentrations. Serum amino acids were measured twice at ~ 1 hour following morning and afternoon oral L-Arg supplementation and twice at ~ 3–5 hours post-L-Arg in the MELAS patients. Following single-dose and 6-week steady state L-arginine dosing in MELAS patients only, there were no statistically significant differences in serum citrulline or glutamate concentrations in MELAS patients when compared to their baseline values, which remained within the normal range. There was a 2- to 3-fold increase in serum ornithine concentrations with the highest value reaching 3.5-fold above the upper normal control range which was highly statistically significant for serum ornithine concentrations following single-dose L-arginine (119.67 ± 20.34 μmol/L; p = 0.0019) as well as following 6-week steady state L-arginine (113.92 ± 26.0 μmol/L; p = 0.0025) compared to baseline values (40.83 ± 11.67 μmol/L; n = 3) (Table 1). There were no improvements in baseline serum lactates for MELAS 1, 2 and 3 respectively (2.2 mM, 4.5 mM, 3.7 mM) respectively following single dose L-arginine (2.9 mM, 6.8 mM, 4.6 mM) or 6 week steady state L-arginine (3.3 mM, 5.4 mM, 2.9 mM).

**Table 1:** Serum ornithine, glutamate, and citrulline concentrations (μmol/L) at baseline and following single-dose and 6 week steady state L-arginine supplementation in MELAS subjects.

Serum Amino Acid (μmol/L)			1 hour post L-Arg (or pre-prandial if baseline)	3–5 hrs post L-Arg (or post-prandial if baseline)	1 hour post L-Arg (or post-prandial if baseline)	3–5 hrs post L-Arg (or post-prandial if baseline)	Mean ± SD (μmol/L) for specific amino acid	Repeated measures ANOVA p-value comparing dose-effect of L-Arg on baseline serum AAs
**Ornithine**	Baseline (no L-Arg)	MELAS 1	68	48	52	49	54.25	
		MELAS 2	39	27 L	35	31	33	
		MELAS 3	42	23 L	42	34	35.25	
	Baseline mean ± SD						40.83 ± 11.67 (n = 3)	
	Single-dose L-Arg	MELAS 1	112	212 H	148 H	94	141.5	
		MELAS 2	60	98	140 H	107	101.25	
		MELAS 3	77	97	130 H	161 H	116.25	
	Single dose mean ± SD						119.67 ± 20.34	p = 0.0019**
	6-wk steady state L-Arg	MELAS 1	113	134 H	180 H	121	137	
		MELAS 2	85	123	135 H	133 H	119	
		MELAS 3	58	72	112	101	85.75	
	Steady state mean ± SD						113.92 ± 26.0 (n = 3)	p = 0.0025**
**Glutamate**	Baseline (no L-Arg)	MELAS 1	28	37	15	18	24.5	
		MELAS 2	89	26	41	44	50.0	
		MELAS 3	36	19	23	20	24.5	
	Baseline mean ± SD						33.0 ± 14.7 (n = 3)	
	Single-dose L-Arg	MELAS 1	49	49	71	24	48.25	
		MELAS 2	45	68	57	44	53.5	
		MELAS 3	44	55	34	39	43.0	
	Single dose mean ± SD						48.2 ± 5.25 (n = 3)	p = 0.0855
	6-wk steady state L-Arg	MELAS 1	56	36	36	44	43	
		MELAS 2	73	46	47	47	53.25	
		MELAS 3	45	41	34	105	56.25	
	Steady state mean ± SD						50.8 ± 6.94 (n = 3)	p = 0.0566
**Citrulline**	Baseline (no L-Arg)	MELAS 1	28	32	32	28	30	
		MELAS 2	24	24	17 L	17 L	20.5	
		MELAS 3	23	23	24	26	24	
	Baseline mean ± SD						24.83 ± 4.80 (n = 3)	
	Single-dose L-Arg	MELAS 1	28	33	31	28	30	
		MELAS 2	13 L	20 L	19 L	24	19	
		MELAS 3	33	38	35	45	37.75	
	Single dose mean ± SD						28.92 ± 9.42 (n = 3)	p = 0.352
	6-wk steady state L-Arg	MELAS 1	23	31	27	33	28.5	
		MELAS 2	21 L	31	24	24	25	
		MELAS 3	23	30	31	34	29.5	
	Steady state mean ± SD						27.67 ± 2.36 (n = 3)	p = 0.506

**Key**: Normal control ranges for Ornithine = 29–125 μmol/L; Glutamate = 14–192 μmo/L; Citrulline = 23–49 μmol/L. H = high; L = low. We used repeated measures ANOVA for the determination of the significance of the differences in mean serum amino acid concentrations in MELAS subjects following single-dose or 6 week steady state L-arginine supplementation compared to mean baseline serum amino acid concentrations with no L-arginine supplementation. P-value < 0.05* statistically significant; P-value < 0.005 ** highly statistically significant. The statistical power for a sample size of 3 patients to detect clinically meaningful differences between baseline (no L-Arginine) compared to L-Arginine dose effects (single-dose or 6-week steady state L-Arginine) on mean serum amino acid concentrations was > 92% for ornithine and < 40% for glutamate and < 10% for citrulline.

### Baseline cerebrovascular reactivity to CO_2_ stimulus and perfusion

In our prior study [21], we compared our three MELAS sibs to our 4 age- and sex-matched study controls and our healthy control adult population (subjects aged 20–30 yrs) which we combined as there was no statistically significant difference between our study controls and healthy control adult population for CVR (n = 18) or CBF values (n = 14). The MELAS siblings had notably reduced CVR values (mean ± SD: MELAS = 0.100 ± 0.026; controls = 0.225 ± 0.049, n = 23; p < 0.001 in frontal cortex; MELAS = 0.18 ± 0.026; controls = 0.258 ± 0.037, n = 23; p = 0.002 in occipital cortex), which correlated inversely with the severity of neurological phenotype and % mutant mtDNA blood (r = - 0.82 for frontal and—0.91 for occipital cortex). In MELAS sibs, mean CVR was reduced to a greater degree in frontal cortex [21]. MELAS sibs also demonstrated global cortical hyperperfusion compared to study controls and normal control adult population (mean ± SD: MELAS = 88.3 ± 16.5 ml/100 g brain/min; controls = 63.2 ± 10.9, n = 18; p = 0.0026 in frontal cortex; MELAS = 102.9 ± 36.2; controls = 64.1 ± 11.6, n = 18; p = 0.001 in occipital cortex) and normative literature values; the degree of hyperperfusion correlated directly with disease severity and % mutant mtDNA in blood (r = +0.85 in frontal cortex; r = +1.0 in occipital cortex for M1 and M2 with no significant correlations when CBFs of all 3 MELAS patients were compared to % mutant mtDNA due to occipital infarct penumbra in M3) [21]. There was reduced perfusion in the occipital region of MELAS 3 where previous infarction had occurred. In MELAS sibs, mean cerebral blood flow was increased more in occipital than frontal cortex for MELAS 1 and 2. Given the more severe neurological phenotypes and % mutant mtDNA in MELAS 3>MELAS 2>MELAS 1 and the strong direct correlation between % mutant mtDNA and cerebral blood flow in frontal cortex (r = +0.85), we would have expected that CBF in MELAS 3 would have similarly been greater than that in MELAS 2 (143.9 ml/100g/min). There was an inverse correlation between CBF and CVR in frontal cortex as expected (r = - 0.99), suggesting that increased resting flows are at the expense of the flow reserve. This relationship was not evident in the occipital cortex, we believe, due to a significant artefact in the measurement of CBF in grey matter likely affected by stroke penumbra and a generalized reduction in the occipital neuropil caused by the ischemic injury in MELAS 3 [21].

### Cerebrovascular reactivity to CO_2_ stimulus following L-arginine supplementation

**Fig 3:**
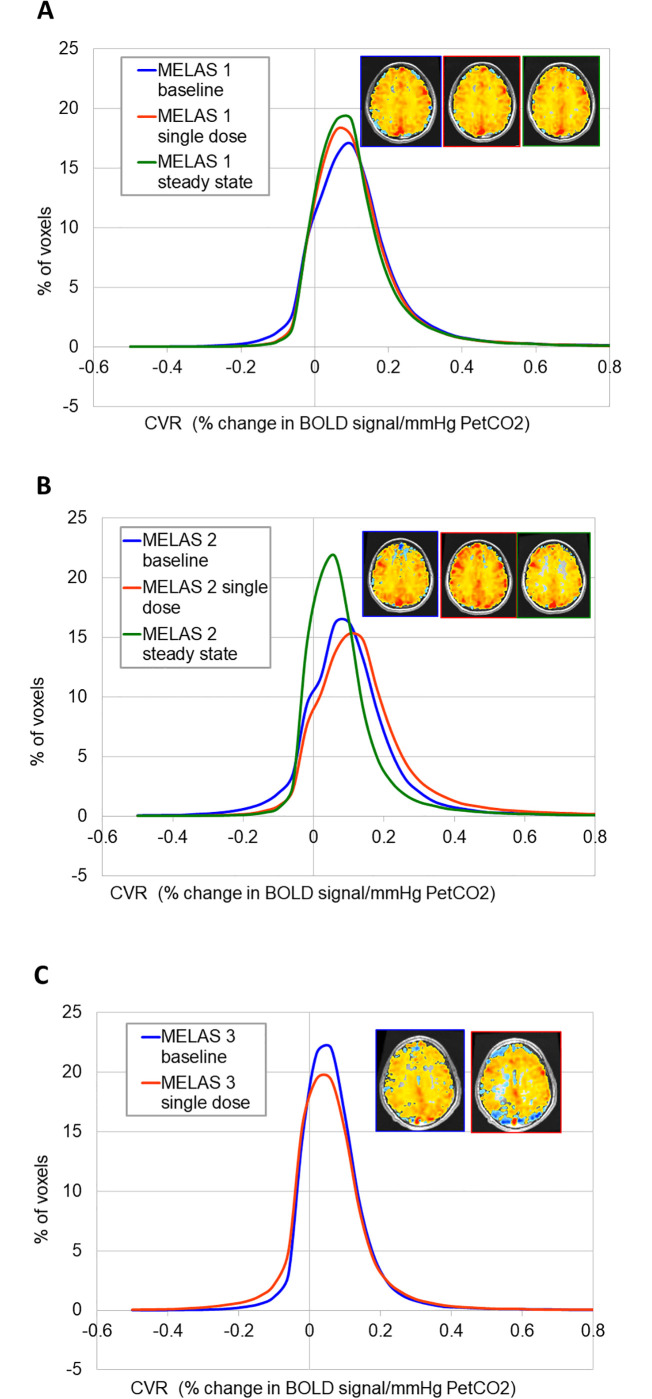
CVR histogram in MELAS patient 1 (A) and MELAS patient 2 (B) at baseline and following single-dose and steady state L-arginine supplementation and in MELAS patient 3 (C) at baseline and following single-dose L-arginine supplementation.

**Fig 4:**
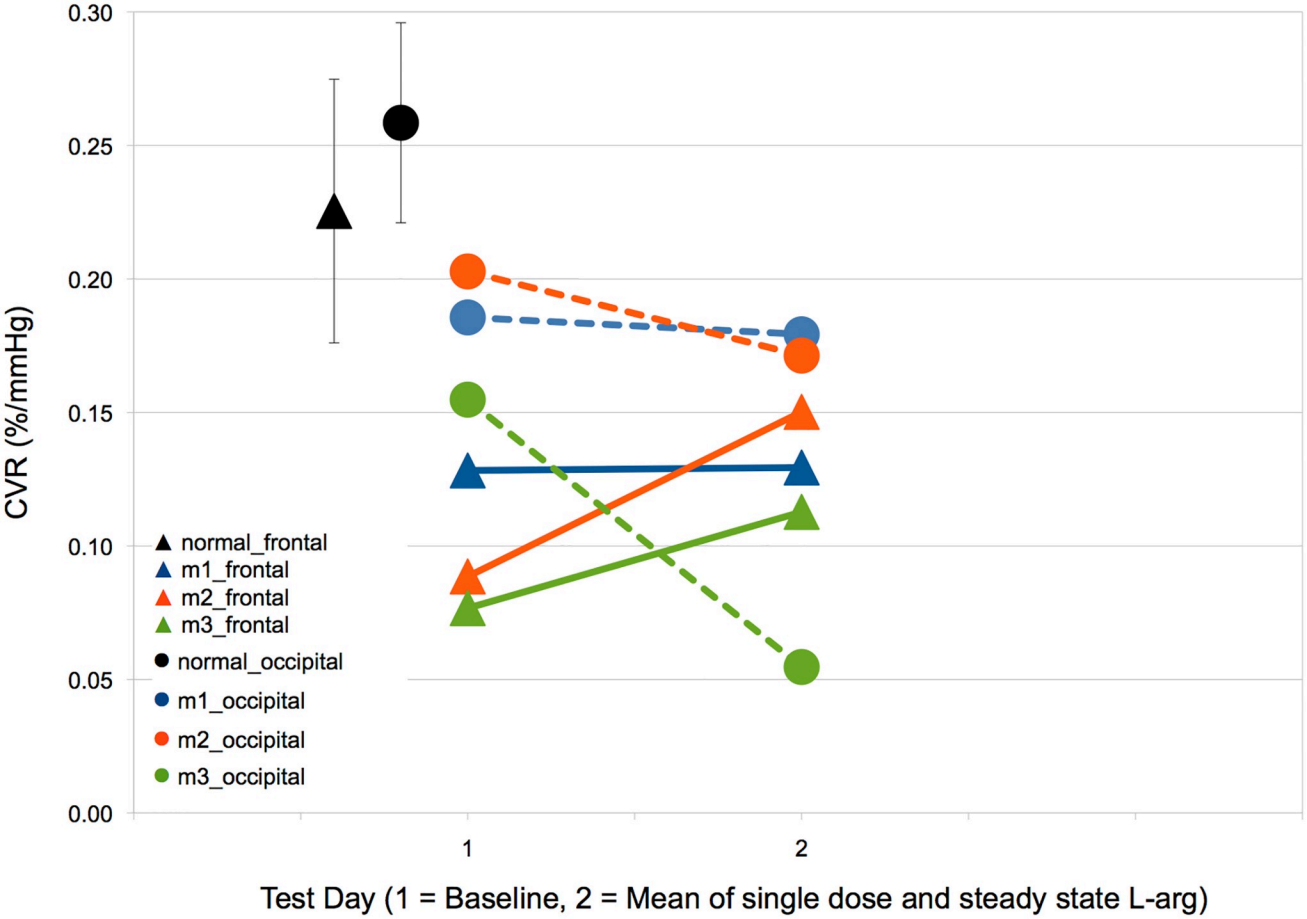
Mean cerebrovascular reactivity (CVR) in frontal and occipital grey matter in MELAS siblings (M1, M2, M3) at baseline and post-combined mean of single-dose and steady-state L-arginine therapy (M3 only single dose).

Comparative CVR histograms for MELAS patients before L-Arg supplementation and following single dose L-Arg, and steady state L-Arg dosing were not significantly different (Fig 3A, 3B and 3C). The CVR study on steady state dosing of L-Arg for MELAS patient 3 was not admissible because of movement artefact. To determine whether there was a differential effect of L-Arg in the occipital versus the frontal grey matter, we calculated the mean CVR in each of these regions in our MELAS patients at baseline and following single dose and steady state L-Arg. On analysis of the neurologically symptomatic MELAS siblings 2 and 3, there was an increase in CVR in the frontal cortex and a corresponding decrease in CVR in the occipital cortex on L-Arg therapy compared to baseline (Fig 4) (Table 2). Asymptomatic MELAS patient 1 demonstrated no major change in CVR in either frontal or occipital cortex. In addition, the MELAS patients showed a narrowing of CVR range in the frontal and occipital cortices from a baseline of 0.08 to 0.20 to a narrower asymptote between 0.11 and 0.17 following L-Arg therapy (with the exception of the occipital lobe CVR in MELAS patient 3 in whom there was only a single-dose L-Arg data point).

**Table 2:** Cerebrovascular reactivity (CVR) and cerebral blood flow (CBF) pre- and post-L-arginine therapy in MELAS patients.

Subject	Baseline	Single-dose L-arginine	Response to single dose compared to baseline	Baseline	6 week steady-state L-arginine	Response to steady state compared to baseline
**CVR (% change/mmHg)**						
*Frontal cortex controls (n = 23) x±SD*	0.225 ± 0.049					
MELAS 1	0.13	0.122		0.13	0.136	
MELAS 2	0.09	0.183		0.09	0.117	
MELAS 3	0.08	0.113			N/A	
Median MELAS 1^st^ to 3^rd^ quartile	0.09 (0.085–0.11)	0.122 (0.117–0.152)		0.11	0.1265	
Mean MELAS ± SD	0.10 ± 0.026	0.139 ± 0.038	39% increase	0.11 ± 0.028	0.126 ± 0.013	15% increase
Mean MELAS vs Total controls *	p < 0.001					
*Occipital cortex controls (n = 23)*	0.258 ± 0.037					
MELAS 1	0.19	0.191		0.19	0.167	
MELAS 2	0.20	0.192		0.20	0.151	
MELAS 3	0.15	0.055			N/A	
Median MELAS 1^st^ to 3^rd^ quartile	0.19 (0.17–0.195)	0.191 (0.123–0.192)		0.195	0.159	
Mean MELAS ± SD	0.18 ± 0.026	0.146 ± 0.078	19% reduction	0.195 ± 0.007	0.159 ± 0.011	19% reduction
Mean MELAS vs Total controls *	p = 0.002					
**CBF (ml/100g brain/min)**						
*Frontal cortex controls (n = 18) x±SD*	63.2 ± 10.9					
MELAS 1	69.91	-		69.91	76.50	
MELAS 2	93.38	-		93.38	66.69	29% reduction
MELAS 3	101.69	-			N/A	
Median MELAS 1^st^ to 3^rd^ quartile	93.38 (81.64–97.53)			81.64	71.60	
Mean MELAS ± SD	88.32 ± 16.48			81.64 ±16.6	71.60 ± 6.94	
Mean MELAS vs Total controls *	p = 0.0026					
*Occipital cortex controls (n = 18)*	64.1 ± 11.6					
MELAS 1	75.42	-		75.42	83.30	
MELAS 2	143.95	-		143.95	91.81	37% reduction
MELAS 3	89.22 (?)**	-			N/A	
Median MELAS 1^st^ to 3^rd^ quartile	89.22 (82.32–116.58)			109.68	87.55	
Mean MELAS ± SD	102.86 ± 36.24			109.68 ± 48.4	87.55 ± 6.01	
Mean MELAS vs Total controls *	p = 0.001					
*MELAS 2 mean frontal & occipital*				118.66 ± 35.7	79.25 ± 17.8	34% reduction

**Key**: CVR = cerebrovascular reactivity (% change/mmHg); CBF = cerebral blood flow (ml/100 g brain/min); N/A = not admissible due to movement artefact; x = mean; SD = standard deviation;

* p–value calculated using unpaired two-tailed Student’s t-test between MELAS and total controls;

** this CBF value likely reflects peri-infarct penumbra and is therefore not a true reflection of the CBF in the occipital grey matter of MELAS 3.

As we go further away from the infarct e.g. 5 mm and 10 mm, the CBF increases to 95.94 and 104.34 ml/100g/min respectively, which may still reflect infarct penumbra.

### Cerebral blood flow following L-arginine supplementation

**Fig 5:**
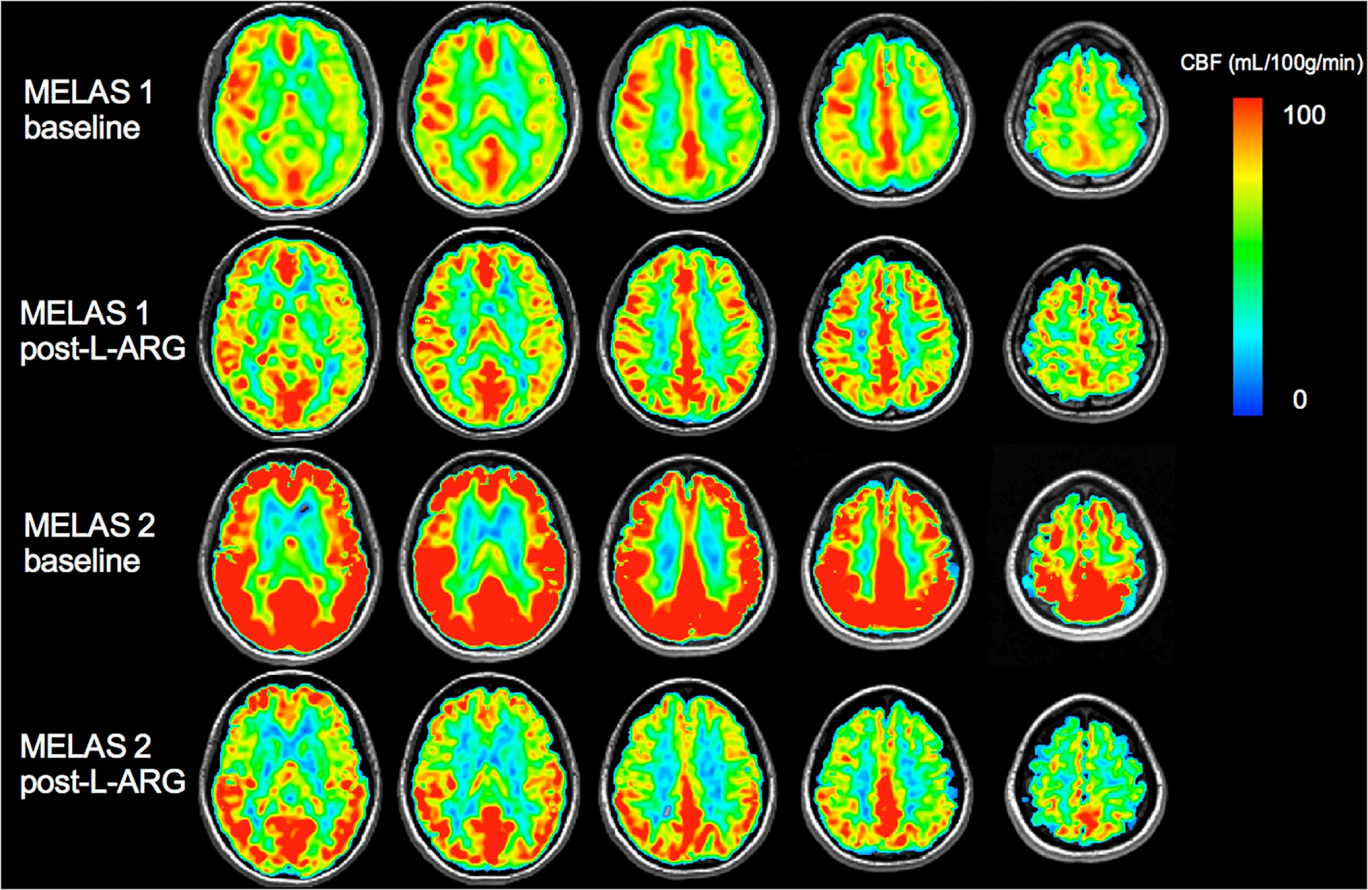
Cerebral blood flow in MELAS siblings (M1, M2) at baseline and post-steady-state L-arginine therapy.

There was no major change in CBF as measured by ASL following 6 weeks of L-Arg therapy in mildly affected MELAS patient 1. There appeared to be a dramatic reduction in the significantly increased baseline CBF in MELAS subject 2 with a 29% reduction in CBF in the frontal cortex and a 37% reduction in CBF in the occipital cortex following 6 weeks of L-arginine (Table 2, Fig 5).

### BOLD fMRI activation in response to an alternating black and white checkerboard pattern

**Fig 6:**
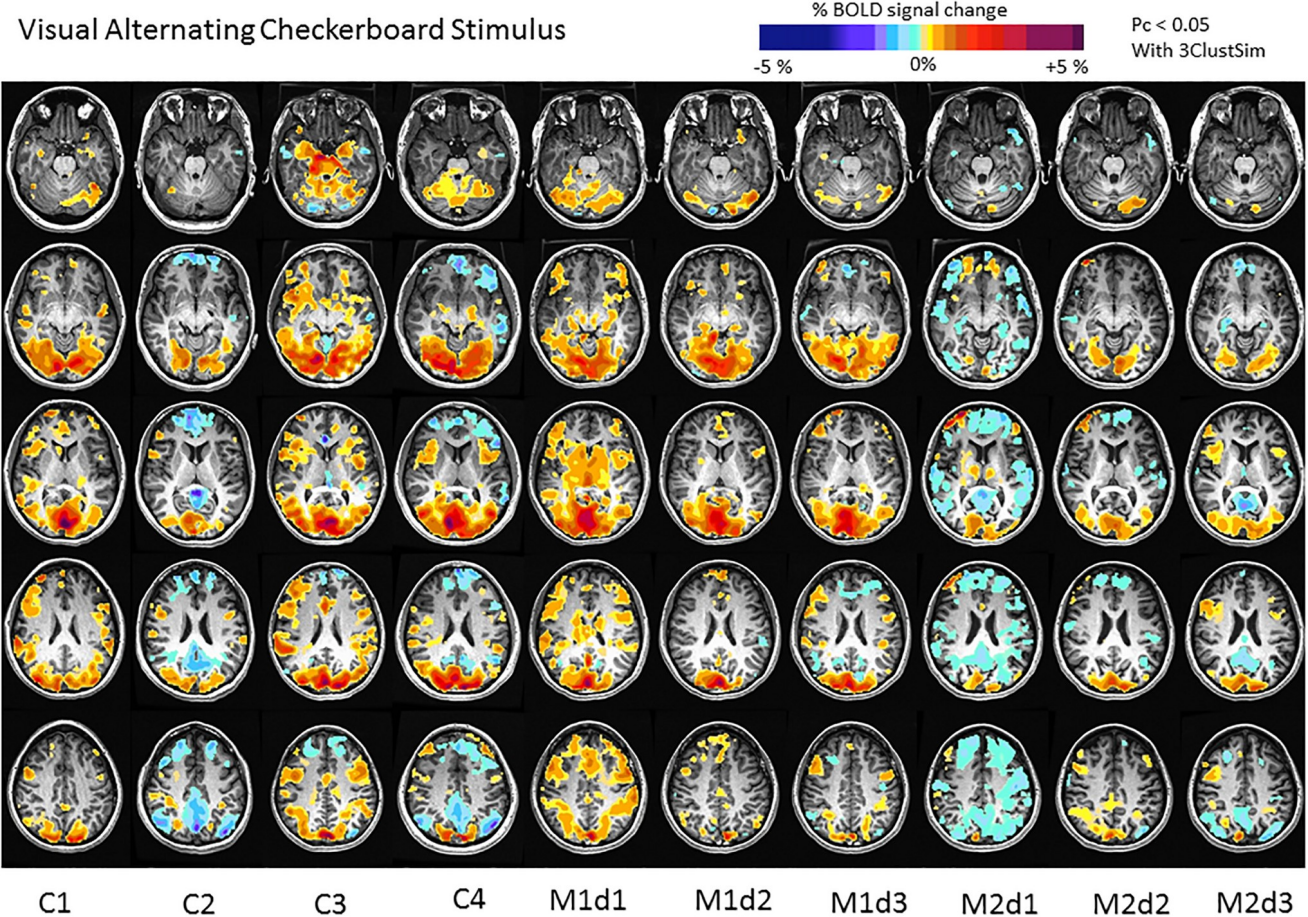
BOLD-fMRI activation in response to alternating checkerboard stimulus in MELAS siblings (M1, M2) at baseline (d1) and post-single-dose (d2) and steady-state L-arginine (d3) therapy compared to controls (C1, C2, C3, C4) at baseline.

There was no major difference in the BOLD fMRI activation to visual stimulus in primary visual striate cortex V1 or in the extrastriate visual cortices (V2 to V5) in mildly affected MELAS patient 1 when compared to our four study controls at baseline or after single-dose or 6 wk steady state L-arginine; all of the values were near to the study control means and within the standard error mean and near to the study control median values being within the 1^st^ to 3^rd^ quartile range of the controls (Table 3, Fig 6). In contrast, in symptomatic MELAS 2 at baseline, compared to study controls, there was a marked reduction in BOLD fMRI activations in response to visual stimulus in visual striate cortex V1 and in extrastriate visual cortices V2, V3, V4 and V5 all of which were well below the 1^st^ quartile of the median of the signal that was seen in the study controls. Following L-arginine, there was a remarkable increase in BOLD fMRI activation in response to visual stimulus in MELAS 2 in the primary visual cortex following single dose L-arginine (though still below the 1^st^ quartile of the median of the study controls) and in the extrastriate visual cortices (though still below the 1^st^ quartile of the median of the study controls) following both single-dose L-Arg with a 2.23 to 2.75-fold increase in activation and following 6 weeks of L-Arg with a 1.92 to 2.03-fold increase in activation. When both the primary visual cortex (V1) and extrastriate visual cortices (V2, V3, V4, V5) activation were averaged together, there was a marked 7.5 fold increase in activation following single dose L-arginine and a 4.37-fold increase in activation following 6 weeks of steady state L-arginine in MELAS 2 suggesting a possible downregulation of the response to L-arginine or alteration in L-arginine metabolism during chronic therapy compared to the single dose trial.

**Table 3:** BOLD fMRI activation in response to alternating checkerboard visual cortex stimulation at baseline in study controls and pre- and post-L-arginine therapy in MELAS siblings.

Subject	Baseline	Single-dose L-arginine	Response to single dose compared to baseline (% increase)	6 week steady-state L-arginine	Response to steady state compared to baseline (% increase)
**V1 visual cortex**					
Study controls x ± SEM (range; n = 4)	1. 46 ± 0.34 (0.42–1.92)				
Study controls median	1.74				
1^st^ - 3^rd^ quartile; (IQ range)	1.07–1.83 (0.76)				
MELAS 1	1.71	1.61		1.26	
MELAS 2	0.31	0.78	2.52-fold	0.27	0.87-fold
**V2 visual cortex**					
Study controls x ± SEM (range; n = 4)	1.08 ± 0.18				
	(0.61–1.38)				
Study controls median	1.16				
1^st^ - 3^rd^ quartile; (IQ range)	0.79–1.36 (0.57)				
MELAS 1	1.18	1.16		0.98	
MELAS 2	0.24	0.66	2.75-fold	0.46	1.92-fold
**V3+4+5 visual cortex**					
Study controls x ± SEM (range; n = 4)	0.76 ± 0.14				
	(0.35–1.01)				
Study controls median	0.83				
1^st^ - 3^rd^ quartile; (IQ range)	0.54–0.97 (0.43)				
MELAS 1	0.65	0.68		0.63	
MELAS 2	- 0.31	0.38	2.23-fold	0.32	2.03-fold
**V1+2+3+4+5 visual cortex**					
Study controls x ± SEM (range; n = 4)	1.01 ± 0.19				
	(0.47–1.31)				
Study controls median	1.13				
1^st^ - 3^rd^ quartile; (IQ range)	0.74–1.28 (0.54)				
MELAS 1	1.06	1.06		0.90	
MELAS 2	0.08	0.60	7.50-fold	0.35	4.37-fold

**Key**: BOLD fMRI activation in response to alternating checkerboard visual stimulus; Cut off for CVR Pc < 0.05 with 3ClustSim for all measurements; x = mean; SEM = standard error mean; IQ = interquartile Broadman visual cortical region masks: V1 = primary visual area and striate cortex located in and around calcarine fissure in the occipital lobe; Extrastriate visual areas: V2 = secondary visual cortex (prestriate cortex) and is the second major area of the visual cortex and the first region within the visual association area. It receives strong feedforward connection from V1 and sends strong connections to V3, V4 and V5; V3 = region located immediately in front of V2; V4 is one of the visual areas in the extrasriate visual cortex and is anterior to V2; V5 = middle temporal visual area and is a region of the extrastriate visual cortex and thought to play a major role in perception of motion.

## Discussion

We have previously demonstrated in our patients with MELAS syndrome (m.3243A>G ^Leu(UUR)^) that CVR between SLEs is reduced and is inversely proportional to the increase in cerebral blood flow [21]. We also unexpectedly found that CVR was more reduced in frontal compared to occipital grey matter. We have previously further shown that baseline cerebral perfusion is increased in patients with MELAS compared to controls and normative cerebral blood flow values reported in the literature [21]. The degree of cerebral hyperperfusion, which translates into a reduction in flow reserve and was inversely proportional to the CVR, was directly proportional to the severity of the neurological phenotype and percentage of mutant mtDNA in blood in our cohort [21]. Increased CBF has also been demonstrated by other investigators using ^133^Xe regional cerebral blood flow studies in a young adult man with MELAS in whom there was generalized hyperperfusion 15 and 26 days after the SLE which was highest in the infarcted areas [36]. The brain continued to be hyperemic with the highest flow in noninfarcted tissue at 4 and 8 months after the SLE. They similarly demonstrated an inverse relationship between resting CBF and CVR. This data supports two different interpretations of MELAS cerebral blood flow physiology. The first hypothesis is that the cortical hyperperfusion in MELAS syndrome may be the result of a normal flow control mechanism responding adaptively in an attempt to compensate for metabolic imbalance resulting from inefficient ATP generation from oxidative metabolism by abnormal non-vascular cerebral mitochondria or may represent a passive response to tissue acidosis or to the accumulation of other intermediary metabolites. In another study, investigators also found generalized cerebral hyperperfusion in a patient following a SLE and found that the hyperemia was accompanied by low cerebral metabolic rate (CMR) for oxygen and oxygen extraction fraction in contrast to preservation of CMR for glucose [37]. This is a pattern consistent with glycolysis to lactate or other intermediate metabolites as it would indicate a reduction in the use of oxygen relative to glucose. These studies support the concept that the metabolic defect and associated hyperemia in MELAS is expressed in cerebral tissue. They may also suggest that the hyperemia is an adaptive response to the limitation in oxidative glucose metabolism and to the reduction of high energy phosphates from the inefficient utilization of oxygen for ATP generation and/or the result of the accumulation of lactic acid or other metabolic intermediates. In our MELAS cohort, using MRS, we did not demonstrate a lactate peak in the occipital cortex. However, this may not have been representative of the other surrounding brain regions, given the limited region of our voxel. Furthermore, MRS would not measure other intermediate metabolites. The second hypothesis is that morphologically abnormal mitochondria in cerebrovascular smooth muscle and endothelial cells may lead to an angiopathy with functional impairment of blood vessel vasodilation in response to an increase in PCO_2_, thereby limiting CVR and supporting a vascular contribution to SLEs. Thirdly, hyperperfusion could be the additive result of both mechanisms which our current studies cannot differentiate. Nonetheless, we hypothesize that perfusion studies could be utilized as a safe, noninvasive and sensitive prognostic marker for patients with MELAS, by which to stratify potential risk for SLEs, with the provision that they are measured in cortical regions of interest, such as the frontal cortex, that are distant from infarcted regions or peri-infarct tissue which may significantly underestimate CBF due to injured neuropil and/or altered vasculature. The markedly exaggerated CBF in the occipital cortex of MELAS patient 2, while of uncertain nature, does raise concern for the capability of matching blood flow in response to energy needs as, provided blood flow is not rate limiting, mutant mtDNA are impaired in their oxygen extraction ability regardless of perfusion rates, and thereby have an ongoing physiological demand for increasing blood flow.

We further demonstrated that the BOLD fMRI activation in response to an alternating checkerboard visual cortex stimulus was within normal limits in unaffected MELAS 1. In contrast, the BOLD fMRI activation to visual stimulus was markedly reduced in the striated (V1) and extrastriate visual cortex (V2 to V5) in symptomatic MELAS patient 2. Notably, the BOLD fMRI activation in MELAS 2 was increased toward normal control values following a single dose and a 6 week steady-state trial of L-Arg therapy. This partial restoration of the BOLD fMRI activation may suggest an improvement in neural activation in the visual cortex and/or in the neurovascular coupling response which includes the complex relationship between neuronal activity and the triggering of the hemodynamic response, followed by the hemodynamic response itself. This may suggest an improvement in the function of the neuronal mitochondria which would have enhanced the neuronal response and/or the cerebrovascular mitochondria which would have enhanced the vascular regulation. The BOLD fMRI BOLD activation was somewhat lower following 6 weeks of steady-state L-Arg than with the single dose in the subject naïve response which may suggest some downregulation of the response through accommodation to the L-Arg or more rapid metabolism of the L-Arg.

Arginine is a dibasic, semi-essential amino acid. Serum arginine concentrations are influenced by dietary intake, endogenous synthesis (kidney and intestine), and protein turnover. Endogenous synthesis occurs though the urea cycle, which converts citrulline to arginine. Arginine is converted to ornithine which has a role in both polyamine and proline synthesis. Arginine itself serves as a precursor for a number of important biochemical reactions [38]. Through the action of endothelial nitric oxide synthase, arginine with NADPH and oxygen are converted to citrulline and nitric oxide with nitric oxide playing a major role in vasodilation. In addition, arginine can be decarboxylated to agmatine, which is a precursor in the polyamine pathway, acts as a neurotransmitter, and inhibits nitric oxide synthase. Arginine is also a precursor for creatine. Furthermore, through a number of reactions, L-arginine following conversion to L-ornithine can be converted to the tricarboxylic acid (TCA) cycle intermediate alpha-ketoglutarate, improving TCA cycle kinetics (anapleurosis) [38] which generates ATP and provides essential reducing equivalents to the mitochondrial respiratory chain. We have shown a statistically significant increase in serum ornithine concentrations with both single dose and steady state oral L-arginine supplementation (Table 1). In addition, arginine can be converted by arginase into ornithine which can then be converted into citrulline by ornithine transcarbamylase. Citrulline can be further converted by two enzymatic steps, namely, argininosuccinate synthetase and argininosuccinate lyase, into arginine [38]. There is a dibasic amino acid transport system in intestinal epithelium and renal tubular cells that transports arginine, lysine, and ornithine. There is still much to be known regarding the intracellular compartmentalization of the aforementioned metabolic pathways, and the cellular transport of arginine [38].

Arginine levels were significantly lower in MELAS patients compared to controls in our study, whereas levels of citrulline and ornithine were similar. Low arginine levels in MELAS are supported by the literature [17]. The etiology of the relative hypoarginemia in MELAS syndrome is not currently known, although a number of theories have been proposed. One group has proposed that the globally increased activity of cytochrome oxidase (COX) in MELAS patients, derived from relatively spared COX activity per mitochondria combined with the proliferation of abnormal mitochondria, consumes nitric oxide and secondarily depletes arginine [39]. Other theories include increased activity of arginase, reduced activity of the cationic amino acid transporter, and/or reduced activity of argininosuccinate synthase [17]. Deficiencies of the other cationic amino acids have not been reported in MELAS syndrome, nor have *consistent* alterations in levels of citrulline or ammonia.

We propose that chronic cerebral hyperperfusion in between SLEs, as we have demonstrated, requires increased production of nitric oxide. Increased nitric oxide synthesis in cells harboring the m.3243A>G ^Leu (UUR)^ mutation has been demonstrated *in vitro* [40]. We believe that there may be a secondary depletion of arginine stores arising from the increased activity of nitric oxide synthase. Significant metabolic stressors such as infection or seizures would necessitate a further commensurate increase in cerebral perfusion from baseline. This would increase nitric oxide requirements and would consequently cause a further drop in arginine levels. Decreased serum arginine levels during SLEs compared to baseline have been demonstrated in previous studies. Energy metabolism may be impaired by arginine deficiency through decreased repletion of the Kreb cycle intermediate alpha-ketoglutarate and decreased creatine production. Hypoarginemia would also contribute to the already decreased CVR that we have previously demonstrated in MELAS patients at baseline. In MELAS, oxygen extraction is impaired, however, oxygen delivery could also become rate limiting. The removal of metabolic intermediates such as free radicals, organic acids and lactate that may contribute toward neuronal toxicity may also depend upon increased perfusion.

We did not demonstrate an overall improvement in interictal CVR in our MELAS patients following L-Arg supplementation; however, on regional analysis there appeared to be an increase in CVR in the frontal cortex (region of most reduced CVR) with a corresponding decrease in CVR in the occipital cortex (region of less reduced CVR). Based on this preliminary finding, it seems as though L-Arg may selectively improve CVR in regions that are most impaired at the expense of less abnormal regions in a manner reminiscent of a vascular-steal. During a SLE, L-Arg may work by “rescuing” regions of the brain at risk by re-routing blood flow from areas with greater reserve. It is important to note that arginine levels were all within the low normal range in our MELAS patients. During a SLE, serum arginine levels will likely decrease and metabolic demand will increase. In these circumstances, CVR may be even more responsive to L-Arg therapy.

Importantly, we demonstrated marked reductions in pre-treatment BOLD-fMRI activation to visual cortex stimulation in affected MELAS patient 2 in the striate (V1) and extrastriate visual regions (V2 to V5) which were increased toward control values following a single dose and 6 wks of L-Arg therapy suggesting an improvement in neuronal activation, neurovascular coupling and/or the hemodynamic response. In MELAS patient 2, we also found a dramatic reduction in cerebral hyperperfusion following 6 wks of L-Arg therapy. This may have been in part related to the role of arginine as a precursor for the energy substrate creatine. In addition, arginine can be converted, through a number of reactions, to the tricarboxylic acid (TCA) cycle intermediate alpha-ketoglutarate, which is critical to improving TCA cycle kinetics (anapleurosis) [38] which generates ATP and provides essential reducing equivalents to the mitochondrial respiratory chain. Energy metabolism may thus be impaired by arginine deficiency documented in MELAS syndrome through decreased repletion of the Kreb cycle intermediate alpha-ketoglutarate and through decreased creatine production. We hypothesize that hyperperfusion may result from energy failure affecting non-vascular cerebral and cerebrovascular mitochondria and/or from vascular dysregulation. Our data may support the concept that L-Arg facilitates metabolic homeostasis and/or vasomotor stability, which could potentially reduce the compensatory hyperperfusion in MELAS syndrome and could potentially play a role in reducing the risk of SLEs. The role of arginine in cellular anaplerosis and overall energy metabolism in MELAS syndrome is yet to be fully elucidated and serves as a key focus for future studies.

Limitations to the present study: We recognize that there were specific limitations to our small pilot study in a single kindred. Firstly, the entire trial was limited to one group of siblings which may limit extrapolation of the results to other individuals with MELAS syndrome with different genetic backgrounds. Secondly, though the diets were relatively constant and similar throughout the study within the same family of siblings, we did not take specific dietary histories which may have influenced amino acid profiles. Thirdly, we did not have the facilities to measure neuronal metabolism with PET scanning which may have further contributed to our understanding of the underlying neuronal and neurovascular pathophysiologic changes with L-arginine supplementation. These factors would ideally be included in future prospective studies in a larger cohort of subjects. Given the variability in clinical phenotypes in individuals with MELAS syndrome, which depends upon the percentage of mutant to wt mtDNA heteroplasmy, comparison of the patients to themselves off and on single dose and steady state L-arginine therapy for changes would seem the most sensitive for detection of changes, as done in this study.

## Supporting information

S1 Checklist CONSORT 2010 checklist of information to include when reporting a randomised trial*. (PDF)

S1 Protocol (PDF)
