# Supplementary material for: L-arginine effects on cerebrovascular reactivity, perfusion and neurovascular coupling in MELAS (mitochondrial encephalomyopathy with lactic acidosis and stroke-like episodes) syndrome
Source: PLoS One. 2020 Sep 3;15(9):e0238224. doi: 10.1371/journal.pone.0238224 (PMC7470264; doi:10.1371/journal.pone.0238224)
Supplement: S1 Checklist — (PDF) [file pone.0238224.s001.pdf]

CONSORT 2010 checklist of information to include when reporting a randomised trial*

Section/Topic	Item No	Checklist item	Reported on page No
**Title and abstract**			
	1a	Identification as a randomised trial in the title	This not a randomized trial; It is a clinical pilot efficacy study
	1b	Structured summary of trial design, methods, results, and conclusions (for specific guidance see CONSORT for abstracts)	Pg. 2 of Clinical Protocol (CP) Pg. 4-5 of submitted paper (SP)
**Introduction**			
Background and objectives	2a	Scientific background and explanation of rationale	Pg. 3 – 4 (CP) Pg. 6 - 8 (SP)
	2b	Specific objectives or hypotheses	Pg. 4 – 5 (CP) Pg. 8 (SP)
**Methods**			
Trial design	3a	Description of trial design (such as parallel, factorial) including allocation ratio	Pg. 7-8 (CP) Pg. 10-11 (SP)
	3b	Important changes to methods after trial commencement (such as eligibility criteria), with reasons	No changes
Participants	4a	Eligibility criteria for participants	Pg. 6-7 (CP) Pg. 9-10 (SP)
	4b	Settings and locations where the data were collected	Pg. 6 (CP) CRC at HSC (SickKids) and UHN

			(Toronto Western Hospital – MRI centre) sites (CP) Pg. 9-10 (SP)
Interventions	5	The interventions for each group with sufficient details to allow replication, including how and when they were actually administered	Pg. 7- 8 (CP) Pg.10-11 (SP)
Outcomes	6a	Completely defined pre-specified primary and secondary outcome measures, including how and when they were assessed	Pg. 8 (CP) Pg.10-14 (SP)
	6b	Any changes to trial outcomes after the trial commenced, with reasons	No changes
Sample size	7a	How sample size was determined	Pg. 8 (CP) Analysis – pilot study to generate estimates of means and SD's to determine adequate samples sizes to detect statistically significant differences in future studies
	7b	When applicable, explanation of any interim analyses and stopping guidelines	Not applicable
Randomisation:			
Sequence generation	8a	Method used to generate the random allocation sequence	Not randomized
	8b	Type of randomisation; details of any restriction (such as blocking and block size)	Not randomized
Allocation	9	Mechanism used to implement the random allocation sequence (such as sequentially numbered containers),	Not applicable

concealment mechanism		describing any steps taken to conceal the sequence until interventions were assigned	
Implementation	10	Who generated the random allocation sequence, who enrolled participants, and who assigned participants to interventions	Pg. 10 (SP) MELAS sibling cohort recruited from Neurometabolic clinic at HSC by clinic nurse; healthy controls recruited by referral and self-selection through posted advertisement at HSC and University of Toronto and screened by PI
Blinding	11a	If done, who was blinded after assignment to interventions (for example, participants, care providers, those assessing outcomes) and how	No blinding
	11b	If relevant, description of the similarity of interventions	Not applicable
Statistical methods	12a	Statistical methods used to compare groups for primary and secondary outcomes	Pg. 8 (CP) Pg.14-15 (SP)
	12b	Methods for additional analyses, such as subgroup analyses and adjusted analyses	Not applicable
**Results**			
Participant flow (a diagram is strongly recommended)	13a	For each group, the numbers of participants who were randomly assigned, received intended treatment, and were analysed for the primary outcome	3 MELAS sibs (2 F, 1 M) 4 Health controls age-

			and sex-matched (see S1. Consort Flow Diagram with paper)
	13b	For each group, losses and exclusions after randomisation, together with reasons	No randomization 4 MELAS sibs, 1 unable to participate; 7 controls, 3 excluded: 1 not meeting inclusion criteria, 1 declined, 1 unable to participate
Recruitment	14a	Dates defining the periods of recruitment and follow-up	Recruitment, clinical testing and follow up in 14 mos from March 2012 to May 2013
	14b	Why the trial ended or was stopped	Trial was completed
Baseline data	15	A table showing baseline demographic and clinical characteristics for each group	See Table 1 in prior PLOS ONE publication of ergometric results in PLoS

			One.2015 May 20;10(5):e0127066.doi:10.1371/journal.pone.0127066.eCollection 2015; PMID 25993630
Numbers analysed	16	For each group, number of participants (denominator) included in each analysis and whether the analysis was by original assigned groups	3 MELAS<br>4 Healthy controls (HC)
Outcomes and estimation	17a	For each primary and secondary outcome, results for each group, and the estimated effect size and its precision (such as 95% confidence interval)	For comparison of MELAS to HC at baseline, the p-value was calculated using unpaired two-tailed Student's t-test; 95 % CI<br>For comparison of MELAS baseline to single dose or steady state L-Arg, p-value was calculated using two-tailed paired t-

			test; 95 % CI; statistical significance set at p-value ≤ 0.05 Pearson's correlation coefficient ® was determined between the % mutant mtDNA blood and the CVR (cerebrovascular reactivity) and between the % mutant mtDNA blood and CBF (cerebral blood flow)
	17b	For binary outcomes, presentation of both absolute and relative effect sizes is recommended	Not applicable
Ancillary analyses	18	Results of any other analyses performed, including subgroup analyses and adjusted analyses, distinguishing pre-specified from exploratory	Not applicable
Harms	19	All important harms or unintended effects in each group (for specific guidance see CONSORT for harms)	No harms or unintended effects in MELAS or HC
**Discussion**			
Limitations	20	Trial limitations, addressing sources of potential bias, imprecision, and, if relevant, multiplicity of analyses	Pg.32-33 (SP)
Generalisability	21	Generalisability (external validity, applicability) of the trial findings	Preliminary pilot data

Interpretation	22	Interpretation consistent with results, balancing benefits and harms, and considering other relevant evidence	Pg.26-33 (SP)
**Other information**			
Registration	23	Registration number and name of trial registry	ClinicalTrials. gov: NCT01603446
Protocol	24	Where the full trial protocol can be accessed, if available	S3: Supplementary With paper
Funding	25	Sources of funding and other support (such as supply of drugs), role of funders	Pg. 3,4 & 33 (SP): UMDF – UMDF had no had no role in the study design, data collection and analysis, decision to publish or preparation of themanuscript Pg. 11 (SP) L-Arginine (NPN80002672) supplied by NOW foods, Bloomingdale, Illinois, USA

*We strongly recommend reading this statement in conjunction with the CONSORT 2010 Explanation and Elaboration for important clarifications on all the items. If relevant, we also recommend reading CONSORT extensions for cluster randomised trials, non-inferiority and equivalence trials, non-pharmacological treatments, herbal interventions, and pragmatic trials. Additional extensions are forthcoming: for those and for up to date references relevant to this checklist, see www.consort-statement.org.
